# Supratentorial extraventricular anaplastic ependymoma in an adult with repeated intratumoral hemorrhage

**DOI:** 10.1007/s10014-013-0146-0

**Published:** 2013-04-02

**Authors:** Naotaka Iwamoto, Yasuo Murai, Yoichiro Yamamoto, Koji Adachi, Akira Teramoto

**Affiliations:** 1 Department of Neurosurgery, Nippon Medical School, 1-1-5 Bunkyo-ku Sendagi, Tokyo, 113-8602 Japan; 2 Department of Diagnostic Pathology, Nippon Medical School, 1-1-5 Bunkyo-ku Sendagi, Tokyo, 113-8602 Japan

**Keywords:** Anaplastic ependymoma, Supratentorial ependymoma, Hemorrhage

## Abstract

We report the case of a 61-year-old man with supratentorial extraventricular anaplastic ependymoma who presented with repeated intratumoral hemorrhage. The patient was admitted with headache. Computed tomography and magnetic resonance imaging showed an enhancing mass with intratumoral hemorrhage in the right temporal lobe. Gross total resection was performed. The tumor was well demarcated from the brain tissue, and showed no continuity with the ventricular system. Histopathological examination revealed the features of anaplastic ependymoma. Therefore, additional radiation therapy and adjuvant chemotherapy were administered. Ten months later, the tumor recurred with hemorrhage in the spinal canal. This case showed rapid malignant progression and repeated intratumoral hemorrhage within a short period of time, both of which are characteristics of anaplastic ependymomas. Close observation of the central nervous system and adjuvant radiotherapy are mandatory, even if the ependymoma presents with repeated intratumoral hemorrhage.

## Introduction

Ependymomas are primary neoplasms of the central nervous system (CNS) that account for about 3–5 % of all adult intracranial gliomas [1, 2]. Ependymomas usually arise from the cells lining the ventricular system and central canal in the spinal cord [3–6]. In a minority of cases, ependymomas arise from the supratentorial parenchyma and show no continuity with the ventricular system. These ependymoma variants are called ectopic, cortical, lobar, or extraventricular ependymomas. Only a few such cases have been reported in the literature [7–15]. In most of these cases, the tumors were difficult to diagnose before surgery. We present a patient with a supratentorial extraventricular anaplastic ependymoma who presented with repeated intratumoral hemorrhage in the brain and spine.

## Case report

**Fig. 1:**
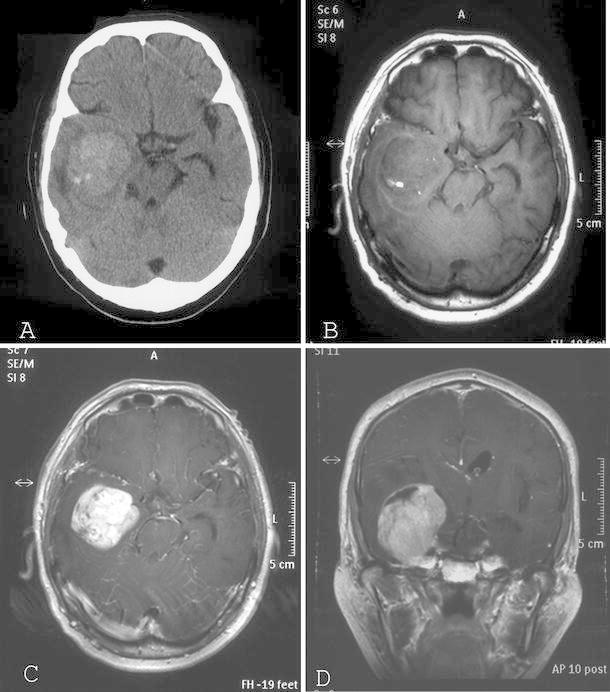
Preoperative imaging studies. **a** Axial-plane CT scan showing a high-density lesion in the right temporal lobe. **b** Axial T1-weighted MR image demonstrating the tumor with heterogeneous intensity located intraaxially and in the extraventricular space. **c** Axial contrast-enhanced T1-weighted MR image demonstrating a strongly enhancing mass. **d** Coronal contrast-enhanced T1-weighted MR image demonstrating a strongly enhancing mass. The tumor occupies the right temporal lobe

**Fig. 2:**
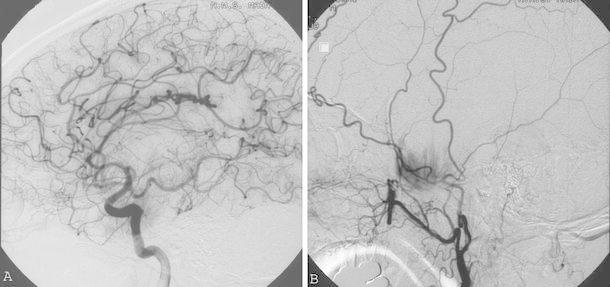
Lateral view of the right internal carotid artery cerebral angiogram (**a**) and lateral view of the right external carotid cerebral angiogram (**b**) performed on December 9, 2008. Early venous filling and tumor staining are observed

A 61-year-old man presented with severe headache on November 30, 2008. A more detailed history revealed that he had been suffering from severe headache of acute onset from 3 days beforehand. Head computed tomography (CT) demonstrated a high-density lesion in the right temporal lobe (Fig. 1a) with a mean diameter of 40 mm. On the magnetic resonance imaging (MRI) performed on December 9, 2008, the lesion was visualized as mixed intensity on T1- and T2-weighted images, and showed strong enhancement following intravenous administration of gadolinium diethylenetriaminepentaacetic acid (Fig. 1b–d). The lesion was surrounded by perifocal cerebral edema. Based on these findings, hemorrhage in the brain tumor was suspected. Cerebral angiography showed that the tumor was supplied by both the internal and external carotid arteries (Fig. 2a, b). Neurological examination did not reveal any neurological deficit during this admission. We planned the operation for January 9, 2009, and the patient was discharged from the hospital.

**Fig. 3:**
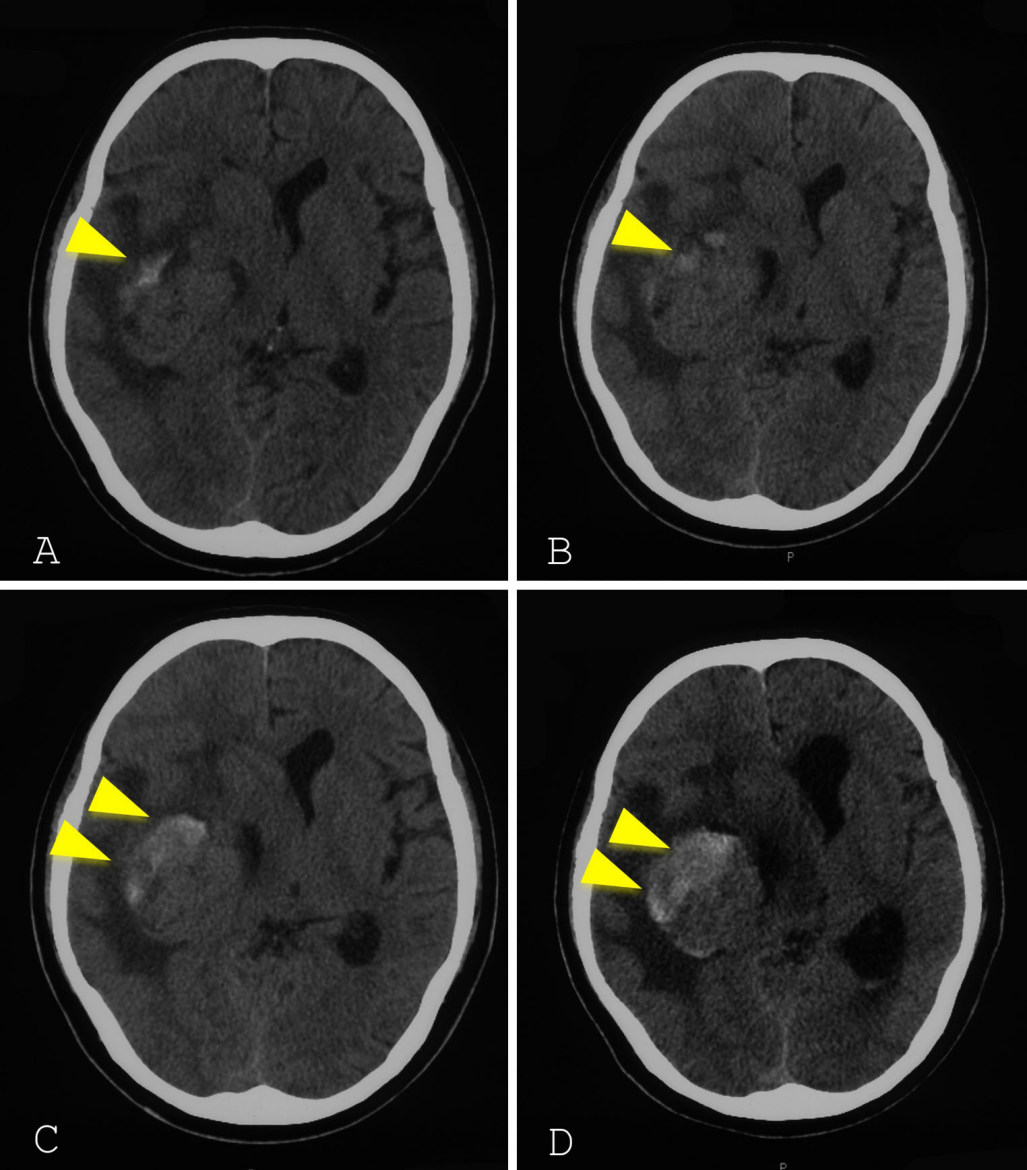
Computed tomography scans obtained on December 26, 2008 (**a**), December 31, 2008 (**b**), January 3, 2009 (**c**), and January 7, 2009 (**d**). A high-density area can be seen in the tumor that gradually expands (*arrow head*). This indicates repeated intratumoral hemorrhage. The perilesional brain edema and displacement of the midline structures deteriorated

The patient presented to the hospital again with severe headache on December 26, 2008. Repeat CT revealed another high-density area within the tumor and more extensive peripheral edema, the findings suggestive of recurrence of the intratumoral hemorrhage (Fig. 3a). The patient was treated conservatively until the operation. During the preoperative period, the patient developed consciousness disturbance. Follow-up CT scans obtained after admission demonstrated another recurrence of the intratumoral hemorrhage (Fig. 3b–d). On January 7, 2009, the patient fell into a coma, and emergent right temporal craniotomy was performed. Intraoperative findings confirmed that the tumor was attached to the dural membrane of the middle fossa, showing no attachment to the ventricular system. The tumor was clearly demarcated from the surrounding brain tissue and gross total resection was performed.

**Fig. 4:**
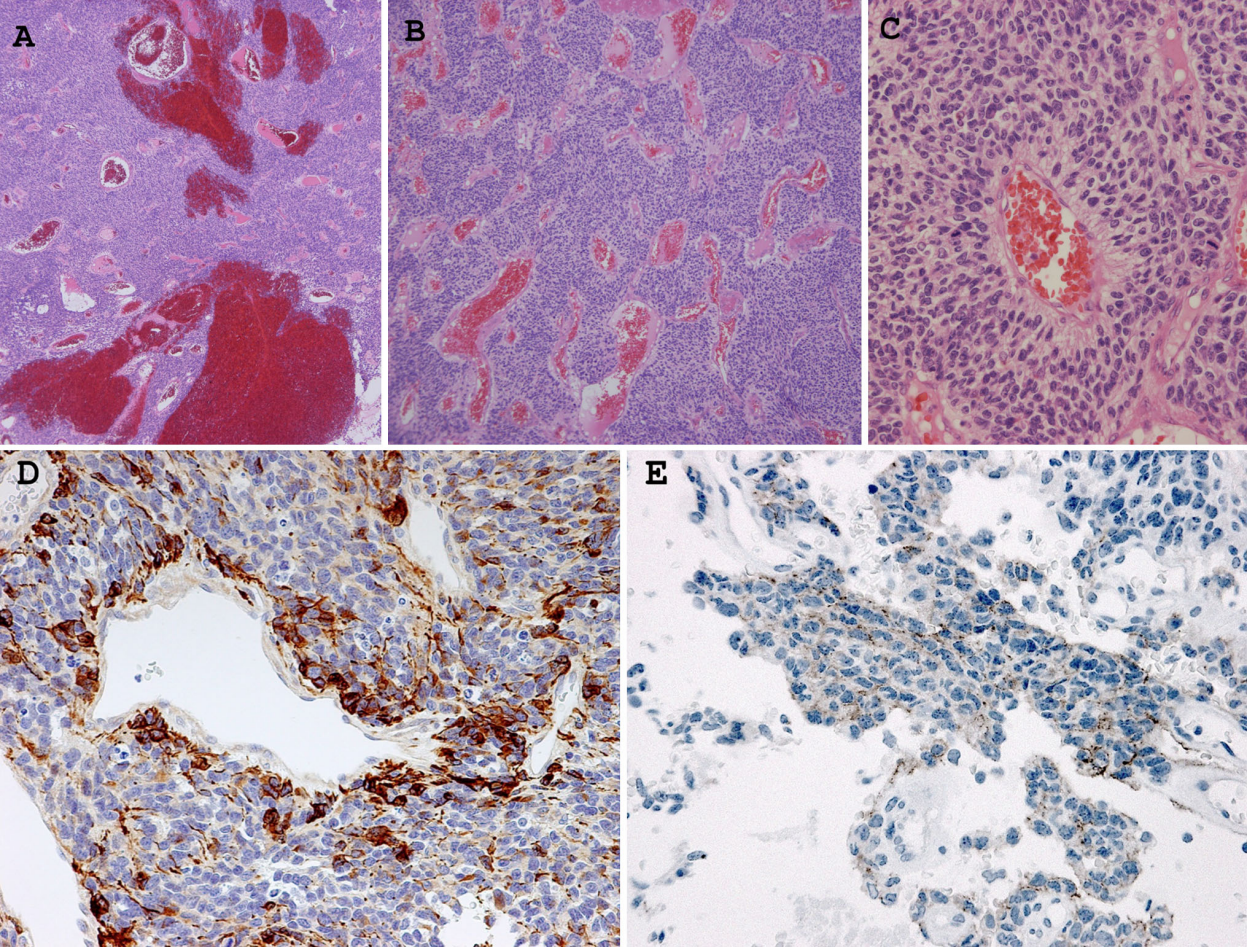
Pathologic micrographs from the surgical specimen. H&E-stained section of the resected tumor (**a** ×40, **b** ×100, **c** ×400). **a** Pathologic micrograph showing the highly cellular tumor. Many hemorrhages were seen. **b** This micrograph shows the highly cellular and well-vascularized tumor. **c** The hyperchromatic nuclei show mild polymorphism. There were numerous perivascular pseudo-rosette formations. Positive immunohistochemical staining for glial fibrillary acidic protein GFAP (**d**) and epithelial membrane antigen (EMA) (**e**) (**d** ×200, **e** ×400). **d** Immunostaining for GFAP showed numerous positive clear cells and positive perivascular tumor cells. **e** Dot-like EMA positivity was seen in the tumor cytoplasm or pericellular area

Histological examination revealed that the lesion was very cellular and well vascularized. Many blood vessels, hemorrhages, and vascular proliferation were seen, but pseudopalisading necrosis was not seen in the specimen (Fig. 4a, b). The nuclei were polymorphic; there were some mitotic figures and numerous perivascular pseudo-rosette formations (Fig. 4c). Immunohistochemical study revealed positive staining of the tumor cells for glial fibrillary acidic protein (GFAP) (Fig. 4d), epithelial membrane antigen (EMA) (Fig. 4e), S-100 protein, and vimentin. However, the tumor showed negative staining for CD34 and bcl2. The MIB-1 labeling index was 10–30 %. The pathological diagnosis was anaplastic ependymoma.

Postoperative MRI demonstrated gross total resection (data not shown). After surgery, focal radiation therapy (60 Gy) and chemotherapy (temozolomide) were administered. The patient showed no neurological deficit after the treatment, and was discharged.

**Fig. 5:**
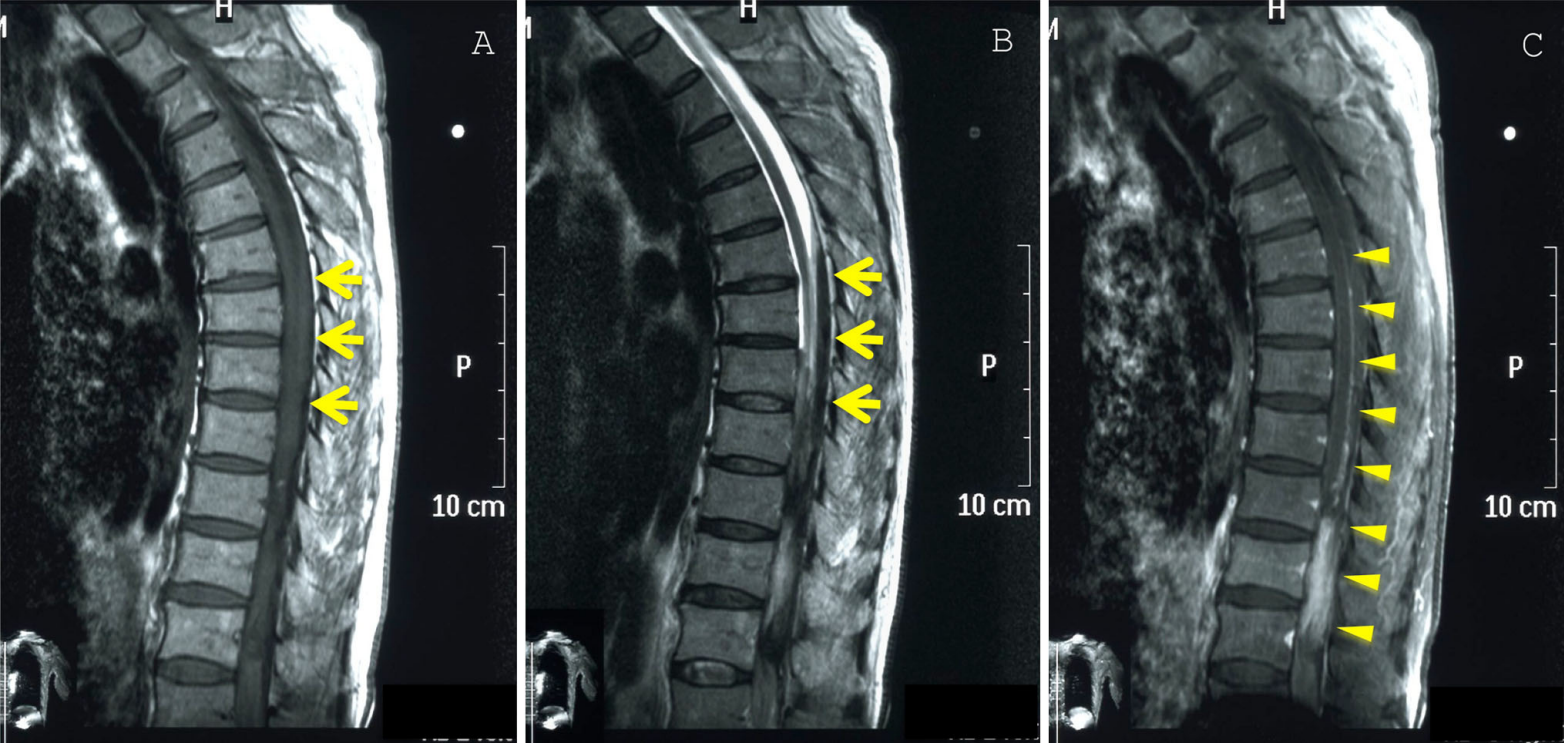
MRI of the thoracic spine. Sagittal T1-weighted MR image (**a**), T2-weighted MR image (**b**), and T1-weighted MR image with contrast enhancement (**c**). **a** Fluid–fluid level was seen in the dorsal side of the spinal cord as a slightly high intensity signal (*arrows*). **b** Fluid–fluid level was seen in the dorsal side of the spinal cord as a low-intensity signal (*arrows*). **c** An intradural extramedullary enhancing mass was seen after intravenous administration of gadolinium diethylenetriaminepentaacetic acid (*arrow heads*). This indicated drop metastases (*arrow heads*) with hemorrhage (*arrows*)

The patient was admitted again with back pain and gait disturbance on November 8, 2009. MRI of the thoracic spine demonstrated a tumor with hematoma in the spinal canal (Fig. 5). A second operation was performed, and histopathological examination revealed recurrence and dissemination of the anaplastic ependymoma.

## Discussion

Ependymomas usually arise from the cells lining the ventricular system and central canal of the spinal cord [3–6]. The clinical courses of patients with intracranial ependymomas can be quite variable [16]. Supratentorial ependymomas in adults are rare CNS tumors that continue to generate considerable controversy with regard to their clinical management [17]. Several negative prognostic parameters have been identified, such as young age, incomplete tumor resection, histological anaplasia, and supratentorial localization [9, 18, 19]. To the best of our knowledge, only 9 case reports of supratentorial extraventricular anaplastic ependymoma, including our present case, have been reported in the literature (Table 1). The mean age of the 9 patients was 40 years, and the male-to-female ratio was 5:4. The tumor was located in the frontal lobe in 3 cases, the parietal lobe in 1 case, the temporal lobe in 2 cases, the temporoparietal lobe in 2 cases, and the parietooccipital lobe in 1 case. In 6 cases, the tumors were contiguous with the brain surface as cortical ependymoma. In 5 of these cases, intratumoral hemorrhage was observed. Hemorrhage was observed in 4 cases of cortical ependymoma.

**Table 1:** Summary of 9 cases of supratentorial extraventricular anaplastic ependymoma

Case no.	Author (year)	Age/sex	Location of ependymoma	Hemorrhage	Staining on angiography	Recurrence
1	Takeshima (2002)	70/F	Frontal	+	No study	–
2	Kojima (2003)	56/F	Temporoparietal	+	No study	Residual lesion
3	Moritani (2003)	50/F	Temporal	–	Hypovascular	Initial location
4	Miyazawa (2007)	32/M	Parietal	+	+	Initial location
5	Toba (2009)	36/F	Frontal	–	No study	–
6	Toba (2009)	18/M	Temporoparietal	+	No study	Initial location, spine
7	Eika (2010)	15/M	Parietooccipital	–	No study	–
8	Flavio (2012)	23/M	Frontal	–	No study	–
9	Present case	61/M	Temporal	+	+	Spine

The case we have reported here presented with at least 5 episodes of intratumoral hemorrhage over a period of 40 days. Only one previously reported case of supratentorial extraventricular ependymoma presented with repeated intratumoral hemorrhage [20]. In that case, 3 episodes of hemorrhage occurred over a period of 2 years. To the best of our knowledge, none of the previously reported cases had repeated intratumoral hemorrhage that occurred within a period as short as that seen in our patient. Intratumoral hemorrhage in supratentorial ependymomas is usually considered a rare event [20, 21], although Romero et al. [22] mentioned that intratumoral hemorrhage is not rare in this tumor. The pathological finding in all of these cases was anaplastic ependymoma. Hemorrhage caused by intracranial neoplasm is usually associated with high-grade malignancy and extensive, abnormal vascularization [23]. Kojima et al. [24] reported that the hemorrhage in the tumor reflects the malignancy grade of the tumor. Ernestus et al. [25] also mentioned that the factor that predisposes the most for bleeding seems to be extensive and abnormal vascularity, and endothelial proliferation or dilated thin-walled vessels were common findings in ependymal tumors with spontaneous hemorrhages. In our case, the histological findings were compatible.

In our present case, the tumor recurred in the spine between the lower thoracic and upper lumbar spinal cord, showing both intratumoral and extratumoral hemorrhage. According to the previous literature, anaplastic ependymomas are characterized by a higher proliferative rate and a greater tendency to disseminate into the cerebrospinal fluid, causing drop metastases. Saito et al. [26] mentioned that anaplastic ependymoma disseminated within the central nervous system without local failure. Our case showed a similar course. Cerebrospinal fluid dissemination of anaplastic ependymoma has been reported to be one of the factors that determine end-of-life prognosis [19, 26, 27].

In conclusion, meticulous MRI follow-up of the CNS is mandatory in adult patients with intracranial anaplastic ependymomas, even after gross total removal of the tumor.

Thus, our experience of this case indicates that supratentorial extraventricular ependymoma with repeated intratumoral hemorrhage should lead to a suspicion of an anaplastic tumor histology. Neurosurgeons should not hesitate to perform a radical initial surgery in such cases. Even after gross total removal of the tumor, adjuvant radiotherapy and close MRI follow-up of the central nervous system are mandatory.
